# Supplementary material for: Bacillus anthracis pXO1 plasmid encodes a putative membrane-bound bacteriocin
Source: PeerJ. 2014 Nov 20;2:e679. doi: 10.7717/peerj.679 (PMC4243335; doi:10.7717/peerj.679)
Supplement: Table S1 [file peerj-02-679-s001.pdf]

Query	Target (NCBI GI or PDB ID)	Species	Identity [%]	Similarity [%]	E-value
**BXA0138**	pXO1-125 (10956372)	Bacillus anthracis	100	100	2.0e-80
	hypothetical protein BCA_A0123 (225871612)	Bacillus cereus	100	100	2.0e-80
	MULTISPECIES: hypothetical protein (445978041)	Bacillus cereus group	100	100	2.0e-80
	hypothetical protein (507028854)	Bacillus cereus	58,7	76,9	2.9e-36
	hypothetical protein BW1_022_00300 (569024440)	Bacillus weihenstephanensis	59,2	75	3.9e-36
	hypothetical protein (487960809)	Bacillus cereus	59,2	75	3.9e-36
	pXO1-117 (499192489)	Bacillus anthracis	47	70,1	1.9e-31
	autotransporter adhesin (fragment 1) (501454838)	Actinobacillus pleuropneumoniae serovar 13 str. N273	30,8	63,5	0,011
	autotransporter adhesin (fragment 2) (501454838)		24,5	59,2	0,26
	autotransporter adhesin (fragment 3) (501454838)		28,3	62,3	0,0058
**BXA0139**	hypothetical protein BCA_A0124 (225871613)	Bacillus cereus	57,3	100	2.7e-71
	hemolysin II (446632776)	Bacillus cereus	68,8	96,8	1.8e-36
	beta-channel forming cytolysin (507010777)	Bacillus cereus	67,7	96,8	3.5e-36
	alpha-hemolysin (487896026)	Bacillus cereus	66,7	95,7	3.6e-36
	hemolysin II (446632779)	Bacillus thuringiensis	67,7	96,8	4.0e-36
	hemolysin II (48926190)	Bacillus anthracis	61,1	92,6	5.0e-11
**BXA0140**	pXO1-123 (10956370)	Bacillus anthracis	100	100	4.3e-26
	hypothetical protein BACI_pCIXO101270 (301068170)	Bacillus cereus biovar anthracis str. CI	100	100	4.3e-26
	MULTISPECIES: hypothetical protein (446649208)	Bacillus cereus group	100	100	4.3e-26
	Thuricin CD (2LA0)	Bacillus cereus	77	84	3,28 Å
	Bacteriocin AS-48 (1E68)	Enterococcus faecalis	30	60	4,79 Å
	Carnocyclin A (2KJF)	Carnobacterium maltaromaticum	24,3	59,4	4,75 Å
